# Trabecular and Cortical Bone of Growing C3H Mice Is Highly Responsive to the Removal of Weightbearing

**DOI:** 10.1371/journal.pone.0156222

**Published:** 2016-05-25

**Authors:** Bing Li, Jeyantt Srinivas Sankaran, Stefan Judex

**Affiliations:** 1 Department of Orthopedics, Tianjin Hospital, Tianjin, 300211, China; 2 Department of Biomedical Engineering, Stony Brook University, Stony Brook, New York, United States of America; Rensselaer Polytechnic Institute, UNITED STATES

## Abstract

Genetic make-up strongly influences the skeleton’s susceptibility to the loss of weight bearing with some inbred mouse strains experiencing great amounts of bone loss while others lose bone at much smaller rates. At young adulthood, female inbred C3H/HeJ (C3H) mice are largely resistant to catabolic pressure induced by unloading. Here, we tested whether the depressed responsivity to unloading is inherent to the C3H genetic make-up or whether a younger age facilitates a robust skeletal response to unloading. Nine-week-old, skeletally immature, female C3H mice were subjected to 3wk of hindlimb unloading (HLU, n = 12) or served as normal baseline controls (BC, n = 10) or age-matched controls (AC, n = 12). In all mice, cortical and trabecular architecture of the femur, as well as levels of bone formation and resorption, were assessed with μCT, histomorphometry, and histology. Changes in bone marrow progenitor cell populations were determined with flow cytometry. Following 21d of unloading, HLU mice had 52% less trabecular bone in the distal femur than normal age-matched controls. Reflecting a loss of trabecular tissue compared to baseline controls, trabecular bone formation rates (BFR/BS) in HLU mice were 40% lower than in age-matched controls. Surfaces undergoing osteoclastic resorption were not significantly different between groups. In the mid-diaphysis, HLU inhibited cortical bone growth leading to 14% less bone area compared to age-matched controls. Compared to AC, BFR/BS of HLU mice were 53% lower at the endo-cortical surface and 49% lower at the periosteal surface of the mid-diaphysis. The enriched osteoprogenitor cell population (OPC) comprised 2% of the bone marrow stem cells in HLU mice, significantly different from 3% OPC in the AC group. These data show that bone tissue in actively growing C3H mice is lost rapidly, or fails to grow, during the removal of functional weight bearing—in contrast to the insignificant response previously demonstrated in female young adult C3H mice. Thus, the attributed low sensitivity of the C3H mouse strain to the loss of mechanical signals is not apparent at a young age and this trait therefore does not reflect a genetic regulation throughout the life span of this strain. These results highlight the significance of age in modulating the contribution of genetics in orchestrating bone’s response to unloading and that the skeletal unresponsiveness of young adult C3H mice to the loss of weight bearing is not genetically hard-wired.

## Introduction

Removal of functional weightbearing during disuse or spaceflight is associated with pathological changes in bone. In human adults, this bone loss is the result of an imbalance between bone formation and resorption, with unloading favoring the latter [1]. Both astronauts as well as earth based rodent models of spaceflight show significant differences in the extent of bone loss between individuals [2–4]. Ground based models, in particular, have demonstrated that genetic makeup is not only a key determinant of bone morphology but may also account for the extent of bone's responsivity to weightlessness [4, 5].

Some of the evidence of genetics playing a role in regulating bone’s response to changes in its mechanical demand stems from studies using inbred strains of mice. In the mouse strain most commonly used in biomedical research, the C57BL/6 (B6) [6], exposure to hindlimb unloading (HLU) for 2wk caused 24% less trabecular bone volume fraction (BV/TV) in the distal femur than in normally ambulating control mice [7]. At the same age (4mo) and anatomical location, female mice from the C3H/HeJ (C3H) strain were largely unaffected by 2wk of unloading [7]. Other inbred strains like BALB/cByJ (BALB) mice lose as much as 60% of their trabecular bone volume fraction after 3wk of hindlimb unloading [8]. Differential changes in bone loss are reflected at the molecular level; unloading decreased transcriptional levels of osteocalcin by 68% and collagen type 1 by 55% in BALB mice, but the magnitude of altered mRNA levels in C3H mice was less than half of those [9].

The genome is not only a key regulator of bone’s catabolic and anti-anabolic response to mechanical unloading but also plays a role in orchestrating the anabolic response to mechanical loading. For mechanical loading, age can significantly modulate the adaptive process within a genetic strain. For instance, young adult C3H mice are only mildly responsive to the application of mechanical loading [10–13], while they actively respond to rest-inserted loading when they are 6mo older [14]. As bone undergoes age related alterations in morphology, density, formation/resorption, or exposure to hormones and cytokines [15–17], a number of factors may account for the differential results. Bone regulates the application of mechanical signals differently from the removal of mechanical signals [4, 18] and it is therefore unknown whether the HLU-resistant character of C3H mice is also influenced by age. Such information may be critical for the development and optimization of diagnostics and treatment interventions based on an individual’s genome. Here we asked the question whether the hindlimbs of young growing C3H mice will be susceptible to the removal of weightbearing.

## Materials and Methods

### Experimental design

All procedures were reviewed and approved by the Institutional Animal Care and Use Committee at Stony Brook University (IACUC). The experimental design of this study has been described, in part, in a previous study which also contains a subset of the μCT data presented here [19]. Thirty-four female, 7wk old C3H/HeJ (C3H) mice, purchased from *The Jackson Laboratory* (Bar Harbor, ME), were weight-matched and assigned to either baseline controls (BC, n = 10), age matched controls (AC, n = 12), or hindlimb unloaded (HLU, n = 12) groups. All mice were weighed prior to group assignment. The study commenced when mice were 9wk old. The duration of the experimental protocols was 3wk. All mice were individually housed and had access to standard rodent chow and water ad libitum. Weight-bearing was removed through hindlimb unloading (HLU), a ground-based model stimulating many physiological effects of microgravity [20–22]. Following euthanasia, femora were fixed in 10% formalin for 24h and stored in 70% ethanol for *ex-vivo* micro-computed tomography (μCT), histomorphometry, and histology. Immediately after harvesting, bone marrow was flushed with Dulbecco's Modified Eagle Medium (DMEM, Gibco, Carlsbad) from both tibiae.

### μCT

High resolution (10μm) micro-computed tomography (μCT) scanning (μCT40, Scanco Medical, Switzerland) was performed on the right distal and diaphyseal femur at 55kV, 145uA with an integration time of 300ms and 1000 projections. A 1500μm long region of interest (ROI), 650μm proximal to the distal physeal-metaphyseal junction, was analyzed to quantify trabecular bone in the metaphysis. For cortical bone in the diaphysis, an 800μm long region centered about the mid-diaphysis was evaluated. An automated algorithm was used to separate trabecular bone from the surrounding cortical shell in the metaphysis [23]. Global thresholding (identical value for all groups) was used to avoid bias in determining μCT outcome variables. To ensure consistency, threshold values were selected visually by comparing raw images to thresholded images by the same operator [8, 23, 24]. Selected thresholds were specific for each anatomical site (trabecular bone in the metaphysis and cortical bone in the diaphysis) as threshold selection may be dependent on specific architectural aspects such as bone thickness (Bouxsein et al., 2010). Trabecular microarchitecture was represented by bone volume fraction (BV/TV), connectivity density (Conn.D), trabecular thickness (Tb.Th), trabecular number (Tb.N), trabecular separation (Tb.Sp), and structure model index (SMI). Total area (Tt.Ar), marrow area (Ma.Ar), and cortical area (Ct.Ar) were calculated as averages over the length of each cortical volume of interest.

### Histomorphometry and histology

Two and nine days prior to completion of the study, all mice were injected (intraperitoneal) with calcein fluorochrome labels (10mg/kg). Following μCT scanning, the undecalcified right femur was embedded in methyl methacrylate [25]. Frontal 8μm thick sections along the longitudinal axis of the femur were cut on a microtome (Leica 2165, Leica Microsystems Inc., IL). The nature of the sections (containing bone’s longitudinal axis vs cross-sectional) may give rise to greater data variability but this choice was necessitated by the number of assays performed on the two tibiae and femurs. Mineralizing surface per bone surface (MS/BS, %), mineral apposition rate (MAR, um/d), and bone formation rate per bone surface (BFR/BS, um^3^/um^2^/d) were measured in two non-consecutive unstained frontal sections of metaphyseal trabecular bone as well as at two locations within mid-diaphyseal cortical bone. Histomorphometric ROIs were defined in analogy to those defined by μCT (Osteometrics Inc., Atlanta, GA). For the calculation of MAR, the lowest MAR value detected across all sections was used for those sections that didn't contain double labels [26].

The left metaphyseal femur was decalcified and embedded in paraffin wax. A single section was stained with 1% toluidine blue (TB) [27] or with tartrate-resistant acid phosphatase (TRAP) [28] to determine osteoblast surface per bone surface (Ob.S/BS, %) and osteoclast surface per bone surface (Oc.S/BS, %) using Osteomeasure software (Osteometrics Inc., Atlanta, GA). Osteoblast and osteoclast surface analysis was not performed in the mid-diaphysis because of technical difficulties.

### Flow cytometry

Bone marrow was flushed from both tibiae with DMEM (Gibco, Carlsbad, CA), supplemented with 2% fetal bovine serum (Gibco, Carlsbad, CA), and filtered through a 70-μm mesh (BD Biosciences, San Jose, CA, USA). Red blood cells were lysed using 1% Pharmlyse (BD Bioscience, San Diego, CA). Total number of bone marrow cells (BMC) pooled from both tibiae was measured using a scepter (EMD Millipore, Darmstadt, Germany). A suspension containing 1×10^6^ BMC from every mouse was stained with APC-conjugated CD90.2 and PE-conjugated SCA1, PE-Cy7-conjugated CD 105, and FITC-conjugated CD34 to detect bone marrow progenitor cells [29, 30]. Cells that were positive for Sca-1 antibody were used to quantify total pooled bone marrow stem cells (SC) [31, 32]. Cells that were positive for Sca-1, CD 90.2, CD 105 and negative for CD34 were quantified to evaluate total pooled osteoprogenitor cells (OPC) [33, 34]. Analysis was performed via fluorescent activated cell sorting (FACS) (FACS Calibur, BD Biosciences) and FACS software (FlowJo; Tree-Star Inc., Ashland, OR). The ratio of stem cells to bone marrow cells (SC/BMC), osteoprogenitor cells to bone marrow cells (OPC/BMC), and osteoprogenitor cells to stem cells (OPC/SC) were reported as a percentage.

### Statistics

Results were reported as mean and standard deviation (mean±SD). Inter-group differences (baseline control, age-matched control and HLU) were tested by one-way ANOVA and Tukey post-hoc tests. Differences in body mass between 7wk and 12wk were tested using paired, two-tailed t-tests. All statistical analyses were performed on GraphPad Prism (5.01 GraphPad Software, Inc.). P-values equal or lower than 0.05 were considered significant.

## Results

### Body mass

At 7wk of age, body mass was not different between any of the groups. At completion of the study (12wk), HLU mice had maintained their 7wk body mass levels but were 12% (p<0.05) lighter than age matched controls.

### Trabecular bone

**Fig 1:**
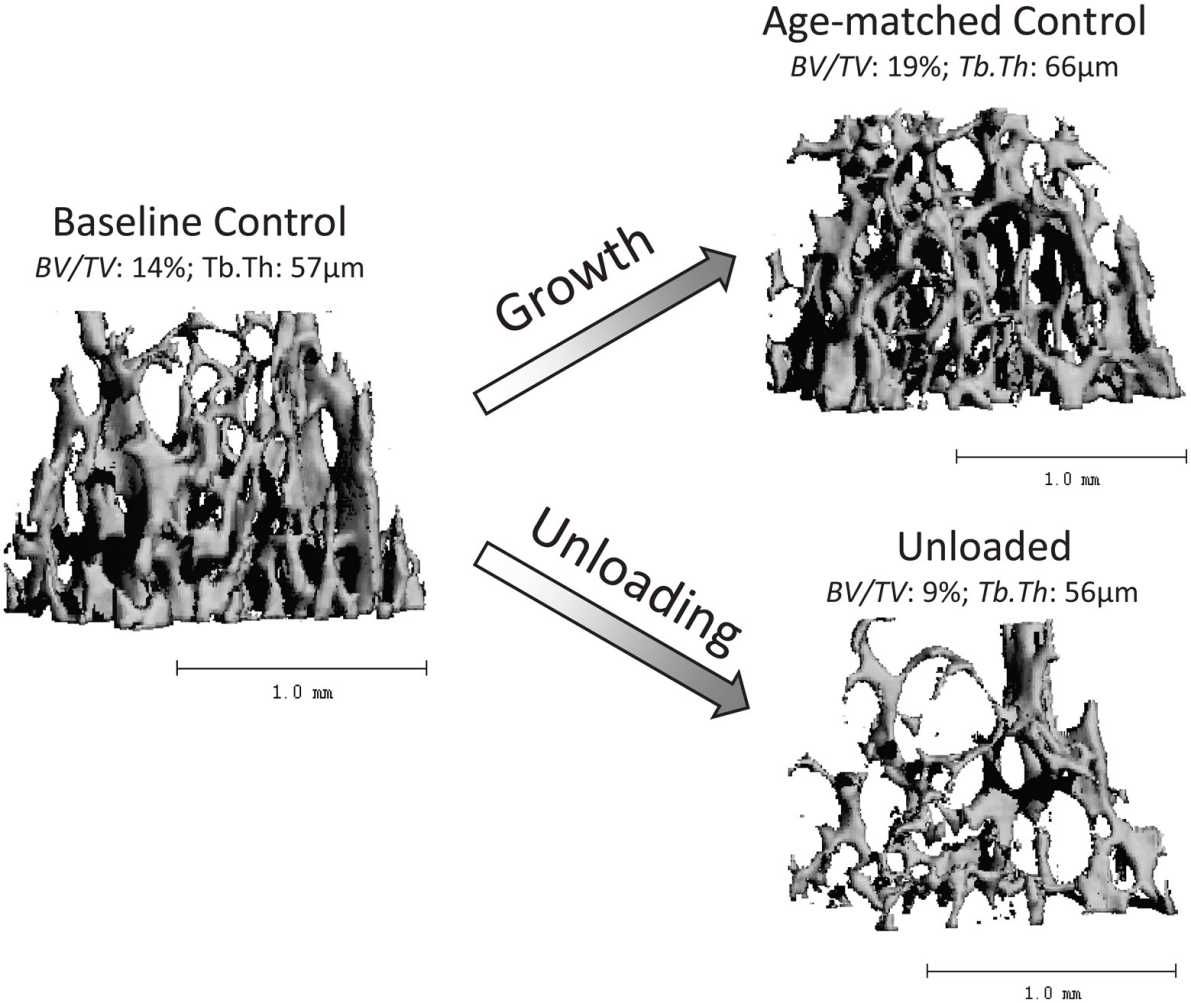
3D tomographies of metaphyseal trabecular bone within the distal femur of a baseline control, age-matched control and unloaded C3H mouse. Normal growth between 9wk and 12wk of age significantly increased trabecular bone volume fraction while unloading significantly decreased it. Shown data present mean group values (see Table 1 for units).

**Fig 2:**
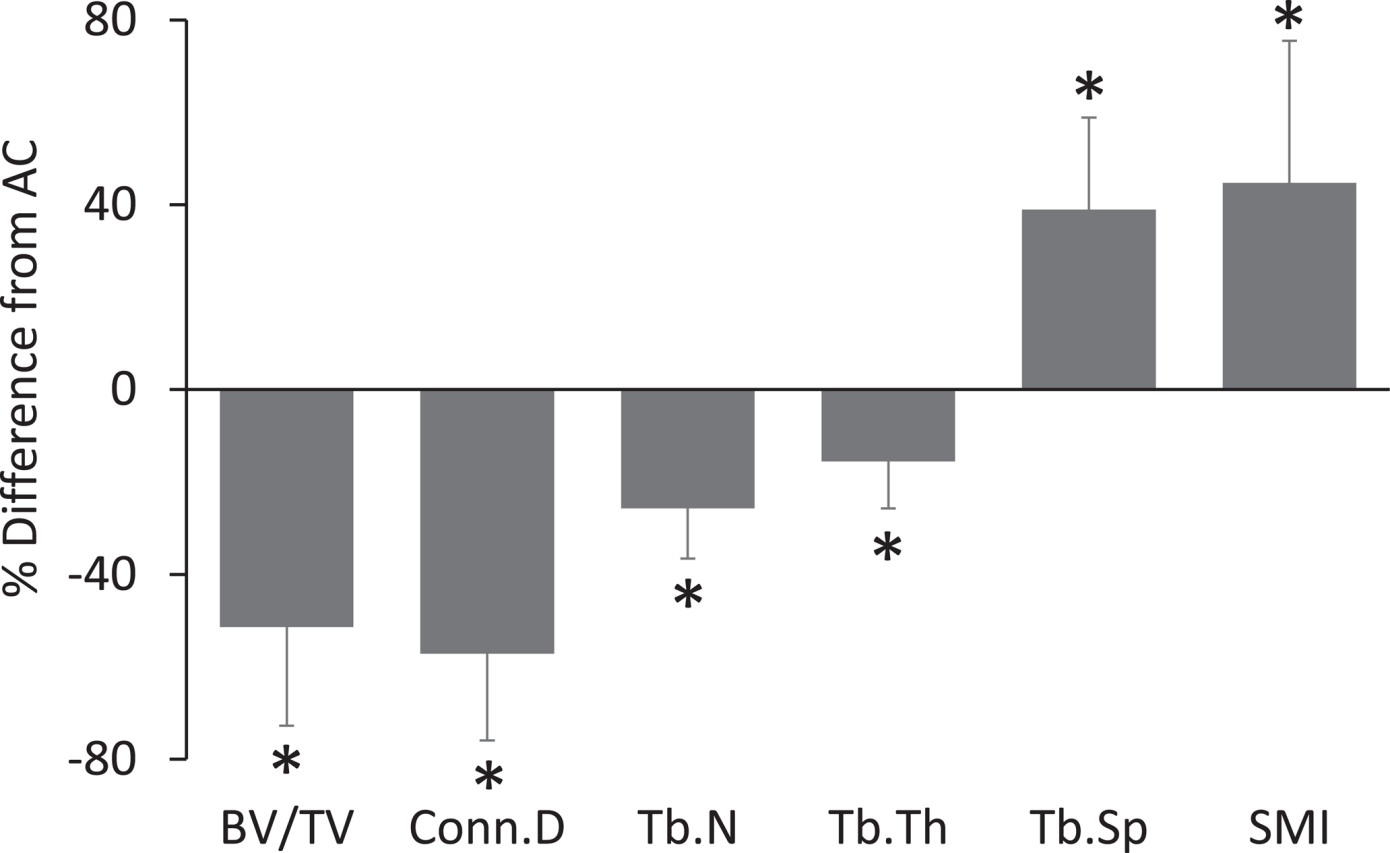
Differences (mean±SD) of trabecular variables in the distal femoral metaphysis of HLU mice when compared to age-matched controls (AC). * = p<0.05 for HLU vs AC

Age related bone growth was observed in the trabecular compartment of normally ambulating C3H mice between 9 and 12 weeks. AC mice had 36% greater BV/TV and 16% greater Tb.Th than baseline control mice (all p<0.05, Table 1, Fig 1). HLU devastated trabecular bone quantity and architecture in the distal femur. When compared to AC, HLU mice had 53% lower BV/TV, 16% lower Tb.Th, 26% lower Tb.N, 58% lower Conn.D, 35% greater Tb.Sp, and 41% greater SMI (all p<0.05; Table 1, Figs 1 and 2). Further, as indicated by comparisons to baseline control mice, HLU resulted in pronounced trabecular degeneration with 35% lower BV/TV, 54% lower Conn.D, 20% lower Tb.N, and 24% greater Tb.Sp (all p<0.05, Table 1, Fig 1).

**Table 1:** Morphological indices of trabecular bone at distal femur metaphysis and the mid-diaphysis in baseline control (BC, n = 10), age-matched control (AC, n = 12) and hindlimb unloaded (HLU, n = 12) C3H mice (mean±SD).

	Index	BC	AC	HLU
**Trabecular**	BV/TV [%]	14.1±3.5	19.1±3.9*	9.0±3.7^†^^‡^
	Tb.Th [μm]	57.1±3.5	66.3±5.1*	55.7±5.2^†^
	Tb.N [1/mm]	4.1±0.60	4.4±0.40	3.3±0.43^†^^‡^
	Tb.Sp [μm]	249±40	224±19	312±46^†^^‡^
	Conn.D [1/mm^3^]	89.2±30.3	98.5±21.1	41.36±17.5^†^^‡^
	SMI [–]	2.2±0.24	1.8±0.32*	2.5±0.35^†^
**Cortical**	Tt.Ar [mm^2^]	1.2±0.08	1.2±0.10	1.2±0.07
	Ma.Ar [mm^2^]	0.50±0.03	0.37±0.04*	0.49±0.05^†^
	Ct.Ar [mm^2^]	0.71±0.06	0.85±0.06*	0.73±0.07^†^

* significant difference between AC and BC.

^†^ significant difference between HLU and AC.

^‡^ significant difference between HLU and BC.

When comparing normally ambulating 12wk old mice to 9wk mice, indices of trabecular bone formation were greater at the later time point, while resorptive activity was not different. Specifically, mineralizing surface and bone formation rate were 34% and 52% greater at 12wk than at 9wk of age (all p<0.05), while MAR, Ob.S/BS and Oc.S/BS were not different at both these time points (Table 2). HLU mice had 42% lower MAR and 40% lower BFR/BS while Ob.S/BS was 39% greater than in age-matched controls (all p<0.05, Table 2). No differences in MS/BS and Oc.S/BS were observed between HLU and AC. When compared to baseline controls, HLU mice had 36% lower MAR, 40% greater MS/BS, and 56% greater Ob.S/BS (all p<0.05). HLU did not alter BFR/BS and Oc.S/BS relative to baseline controls. The referent bone surface (BS) was 22% and 26% lower in HLU than in AC and BC mice (all p<0.05).

**Table 2:** Cellular parameters of bone formation and resorption measured at the distal metaphysis and the mid-diaphysis of the femur in baseline control (BC, n = 10), age-matched control (AC, n = 12) and hindlimb unloaded (HLU, n = 12) C3H mice (mean±SD).

	Index	BC	AC	HLU
**Trabecular**	MS/BS [%]	15.7±4.2	21.1±3.3*	22.1±4.8^‡^
	MAR [μm/d]	1.5±0.18	1.6±0.32	0.94±0.21^†^^‡^
	BFR/BS [mm^2^/(mm·d·10^3^)]	0.23±0.07	0.35±0.10*	0.21±0.06^†^
	Ob.S/BS [%]	26.6±4.3	29.9±9.5	41.4±9.5^†^^‡^
	Oc.S/BS [%]	18.0±5.2	17.1±7.1	13.3±6.0
**Cortical**	Ec MS/BS [%]	38.3±10.0	37.5±9.0	27.4±12.4^†^^‡^
	Ec MAR [μm/d]	2.1±0.67	2.2±0.65	1.1±0.74^†^^‡^
	Ec BFR/BS [mm^2^/(mm·d·10^3^)]	0.82±0.40	0.83±0.31	0.39±0.33^†^^‡^
	Ps MS/BS [%]	20.7±11.5	27.0±11.5	17.6±10.3^†^
	Ps MAR [%]	1.7±0.71	1.7±0.98	1.2±0.98
	Ps BFR/BS [mm^2^/(mm·d·10^3^)]	0.39±0.25	0.47±0.27	0.24±0.23^†^

* significant difference between AC and BC.

^†^ significant difference between HLU and AC.

^‡^ significant difference between HLU and BC.

### Cortical bone

**Fig 3:**
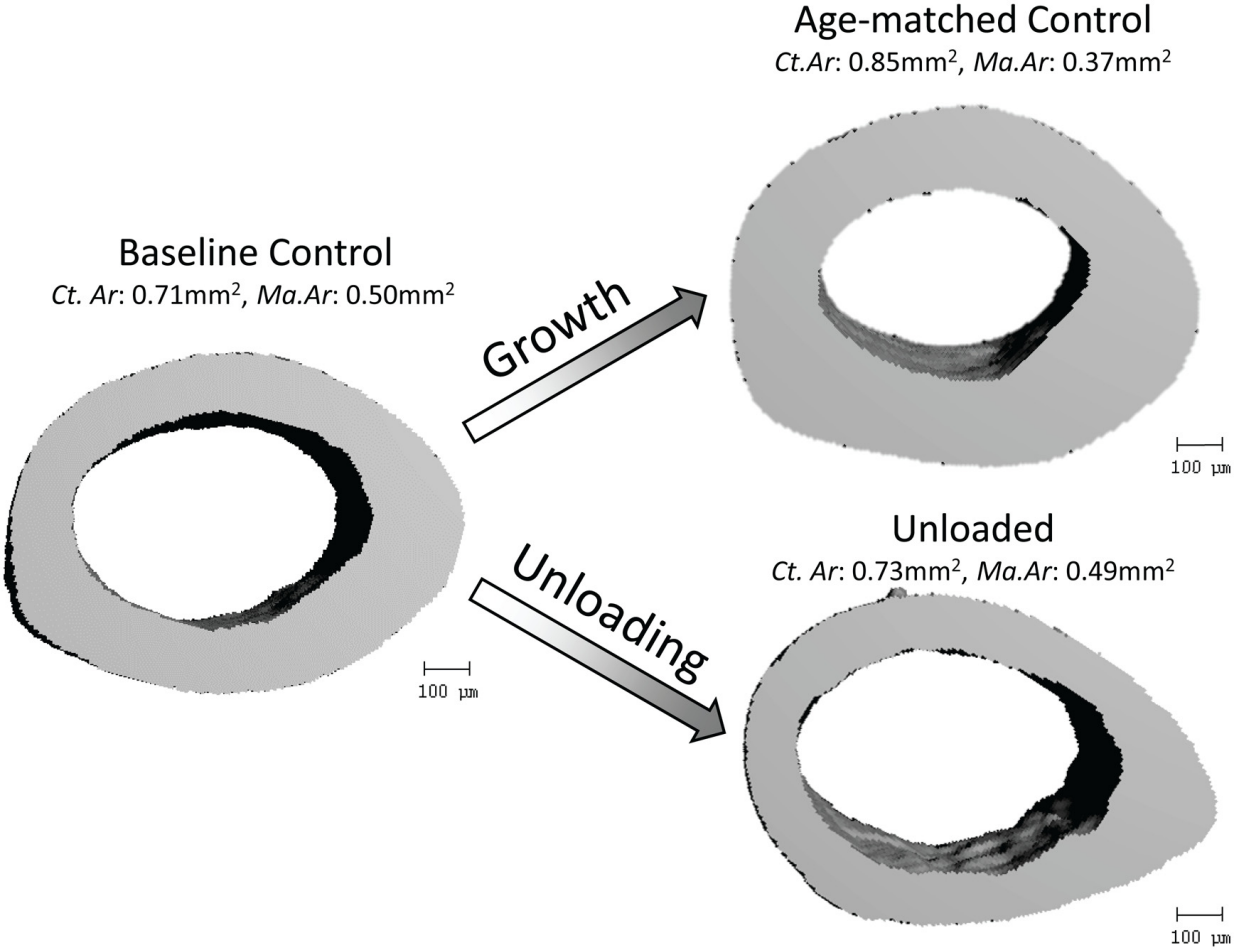
3D tomographies of mid-diaphyseal cortical bone in the femur of a baseline control, age-matched control and unloaded C3H mouse. Normal growth between 9wk and 12wk of age significantly increased cortical and decreased marrow areas while unloading suppressed these changes. Shown data present mean group values (see Table 1 for units).

In normally ambulating control mice, cortical bone of the diaphysis expanded between 9wk and 12wk controls. 12 wk old mice had 20% greater Ct.Ar and 26% lower Ma.Ar than 9wk old mice (all *p<0*.*05*). Unloading inhibited normal growth at the endocortical surface, resulting in 24% greater Ma.Ar and 14% lower Ct.Ar (all *p<0*.*05*) in HLU mice versus age-matched controls. Tt.Ar was unaffected by HLU. Cortical indices were not different between HLU and BC mice (Table 1, Fig 3).

Dynamic indices of cortical bone formation were not different between 9wk and 12wk of age at both the endocortical and periosteal surfaces (Table 2). On the endocortical surface, HLU mice had 27% lower MS/BS, 47% lower MAR, and 53% lower BFR/BS than age-matched controls (all p<0.05). On the periosteal surface, HLU mice had 35% lower MS/BS and 49% lower BFR/BS than AC mice (all p<0.05). When compared to baseline control mice, HLU mice had 29%, 43%, and 52% lower MS/BS, MAR and BFR/BS (all p<0.05, Table 2) at the endocortical surface (Table 2). Indices of bone formation were not statistically different between HLU and BC at the periosteal surface even though mean values for MS/BS, MAR and BFR/BS were 15%, 29% and 38% lower in HLU than BC.

### Bone marrow progenitor cells

The number of total bone marrow cells (BMC), stem cells (SC) and osteoprogenitor cells (OPC) were not different between HLU and AC mice. There was also no difference in the ratios of SC/BMC and OPC/BMC between the two groups. In contrast, the ratio of OPC/SC was different between HLU and AC mice; only 2% of the SC were of OPC lineage in HLU mice while in AC mice, 3% of the SC were enriched toward the OPC lineage (p<0.05, Table 3).

**Table 3:** Bone marrow cells (BMC), stem cells (SC), osteoprogenitor cells (OPC), and proportions of SC/BMC, OPC/BMC, and OPC/SC (mean±SD) for AC (n = 12) and HLU mice (n = 12).

Index	AC	HLU
**BMC (count)**	1.09·10^7^±4.04·10^6^	9.12·10^6^±4.10·10^6^
**SC (count)**	7.28·10^5^±2.92·10^5^	6.60·10^5^±2.80·10^5^
**OPC (count)**	2.20·10^4^±1.01·10^4^	1.57·10^4^±8.99·10^3^
**SC/BMC (%)**	6.6±0.86	7.8±2.08
**OPC/BMC (%)**	0.21±0.07	0.18±0.06
**OPC/SC (%)**	3.1±0.92	2.3±0.47^†^

^†^ significant difference between HLU and AC.

## Discussion

Based on data from adult mice, the skeleton of female C3H mice is considered largely unresponsive to mechanical unloading. In contrast, here, we show that in young skeletally immature female C3H mice, unloading resulted in both trabecular and cortical bone structures vastly different from age-matched ambulatory controls. During the 3wk experimental phase, trabecular and cortical tissue was added in normal ambulatory control mice; twelve-week-old control mice had greater trabecular bone volume and cortical area than 9wk old mice, paralleled by greater bone formation rates in trabecular bone. HLU suppressed this age-related bone growth particularly in the trabecular compartment, resulting in an overall loss of bone tissue compared to baseline controls. No differences in trabecular resorptive activity were detected at the endpoint of the study but transient differences may have been missed. In the cortical compartment, appositional growth was suppressed by HLU but relative to baseline controls, no overall loss was observed. Taken together, these data demonstrate that the trabecular and cortical femur of young female C3H mice is highly sensitive to the removal of weight bearing and that unloading-induced trabecular bone loss was primarily due to suppressed bone formation rather than an increase in resorptive activity.

Previously, we found that female young adult (16wk) C3H mice were largely unresponsive to mechanical unloading [8]. After two weeks of unloading, trabecular bone volume fraction was unaffected in both the metaphysis and the epiphyseal regions of the femur. Cortical area of the femoral metaphysis and diaphysis were also protected from the catabolic effects of unloading at this age [8]. In great contrast, here, both the cortical and trabecular compartments of 9wk old C3H mice were highly susceptible to hindlimb unloading, directly challenging the hypothesis that an individual’s susceptibility to disuse is uniquely and statically embedded within the genetic code. An inherent limitation of the comparison between these two studies is the different unloading duration. Using in vivo μCT scanning, we found previously that over the course of three weeks of hindlimb unloading, the rate of bone loss was similar during the first, second, and third week in genetically heterogeneous mice [4]. Extrapolated to our growing C3H mice, a loss of 53% in BV/TV over 3wk would translate to 35% over 2wk, still a much greater loss than the 8.5% in adult C3H [8]. This extrapolation is corroborated by data from adult male C3H mice in which both 2wk and 3wk HLU protocols did not induce significant bone loss [7].

That genetic polymorphisms strongly interact with age in determining the amount of bone loss during unloading raises the question which of the large number of physiologic changes during late-stage growth accounts for the differential mechanosensitivity between skeletally immature and mature C3H mice. Rates of bone growth and circulating levels of systemic factors are significantly different between a growing versus a more quiescent skeletal stage. Here, dynamic trabecular bone formation rates increased between 9 and 12wk of age, an age after which they begin to decline [16]. Similarly, circulatory levels of insulin like growth factor 1 (IGF1), a potent stimulator of bone formation, gradually decline between 1 and 10 months in C3H mice [35]. Even if considering only differences in these two variables between young and adult C3H mice, it may not be surprising that HLU which exerts its influence in C3H mice primarily by suppressing bone formation is more effective at a stage during which the skeleton actively forms tissue.

Differences in the regulation of body mass by unloading between young and older mice are unlikely to explain differences in their skeletal responses. While weight loss has the potential to exacerbate bone loss [36] and a decline or failure to gain body mass is common during hindlimb unloading [5, 37, 38], we observed comparable differences (changes) in body mass between HLU mice and controls in both C3H studies. Further, across a large sample of genetically heterogeneous mice, an individual’s change in body mass was not correlated with the change in trabecular properties during HLU [39]. Similar disassociations between body and bone mass were apparent when comparing different inbred strains. Young adult C3H mice lost significant amounts of body mass during 2wk of HLU without significant bone loss [8]. In contrast, 2wk of HLU in B6 mice had no effect on body weight but caused significant bone loss [8]. Regardless of the influence of weight, it is clear that the number of physiologic differences between a growing and adult mouse is large, including a potentially differential stress response. Thus, the identification of the factors underlying the interaction between the genetic modulation of disuse and age will require significant future efforts.

Genetic control and the molecular response of bone to mechanical *loading* is distinct from *unloading* [4, 5]. Despite these differences and similar to their response to unloading, young adult C3H mice were unresponsive to mechanical loading [5, 7, 10, 12]. And similar to our data presented here, age was found to be a factor interacting with the ability of the C3H skeleton to respond to mechanical loading. When using older C3H mice (36wk of age), mechanical stimuli were perceived as anabolic as indicated by increased periosteal bone formation rates [40]. At that age, basal bone formation rates were lower than at young adult (4mo) levels. Thus in contrast to our study, lower rather than higher basal osteoblast activity led to an increased response to a change in the mechanical environment, consistent with the differential effects of the application versus removal of mechanical stimuli on bone formation rates. Taken together, it is apparent that the adjustment of bone formation to a new mechanical environment is strongly age dependent in C3H mice.

Unloaded mice had less osteoprogenitor cells than controls, consistent with previous studies demonstrating suppressed osteoblastic differentiation and osteogenic gene expression following HLU [41–43]. While we did not detect differences in SC/BMC here, we previously reported an unloading induced 25% reduction in SC/BMC in B6 mice [33]. This difference may in part reflect the different usage of stem cell markers. Further, lower apoptosis levels inherent to C3H osteoblasts [44] may suppress apoptosis of bone marrow progenitors, also contributing towards the incongruent results between our studies in B6 and C3H mice. The differential response to HLU between these two strains is further emphasized by greater Ob.S/BS in C3H HLU mice (mediated by lower BS) but lower Ob.S/BS in B6 HLU mice when compared to age-matched controls. It should also be noted that OPC identified by flow cytometry may have included other native bone marrow cells as the four surface markers to characterize osteoprogenitors in the bone marrow were not necessarily unique to this cell type [45, 46]. Lastly, our flow cytometry only provided data on the number and proportion of enriched bone marrow cells, and cells will need to be cultured to elucidate function and activity in the future [47].

In summary, we demonstrated that bone of growing female C3H mice is susceptible to mechanical unloading, primarily as a result of suppressed bone formation. Compared to previous data on unloading in young adult C3H mice, our results also indicate a strong interaction by which age influences the genetic control of anabolic activity reduced by unloading. Extrapolated, these data suggest that the susceptibility of an individual to disuse is not entirely predetermined by genetic make-up but dependent on age and associated physiologic factors that may include basal bone formation rates. By extension, countermeasures for disuse osteoporosis may take advantage of this interaction by selectively manipulating these factors.
